# Canola Responses to Drought, Heat, and Combined Stress: Shared and Specific Effects on Carbon Assimilation, Seed Yield, and Oil Composition

**DOI:** 10.3389/fpls.2018.01224

**Published:** 2018-08-30

**Authors:** Raed Elferjani, Raju Soolanayakanahally

**Affiliations:** Saskatoon Research and Development Centre, Agriculture and Agri-Food Canada, Saskatoon, SK, Canada

**Keywords:** photosynthesis, mesophyll conductance, water-use efficiency, nitrogen isotopic discrimination, saturated fatty acids, seed protein, *Brassica napus* L.

## Abstract

Photosynthetic assimilation is remarkably altered by heat and drought, and this depends on the individual or combined occurrence of stressors and their respective intensities and durations. Abiotic stressors may also alter the nutritional quality and economic value of crops. In this controlled greenhouse study, we evaluated the response of *Brassica napus* L., from flowering to seed development, to two temperature and water treatments and a combination of these treatments. The diffusional limitations of stomatal conductance and mesophyll conductance on photosynthesis, as well as resource-use efficiency (particularly water and nitrogen), were assessed. In addition, the effects of stressors on the seed fatty acid content and composition and the total protein content were examined. The results showed that the reduction in the net photosynthetic assimilation rate was caused by combinations of heat and drought (heat + drought) treatments, by drought alone, and, to a lesser extent, by heat alone. The stomatal conductance decreased under drought and heat + drought treatments but not under heat. Conversely, the mesophyll conductance was reduced significantly in the plants exposed to heat and heat + drought but not in the plants exposed to drought alone. The carboxylation efficiency rate and the electron transport rate were reduced under the heat treatment. The seed yield was reduced by 85.3% under the heat treatment and, to a lesser extent, under the drought treatment (31%). This emphasizes the devastating effects of hotter weather on seed formation and development. Seed oil content decreased by 52% in the plants exposed to heat, the protein content increased under all the stress treatments. Heat treatment had a more deleterious effect than drought on the seed oil composition, leading to enhanced levels of saturated fatty oils and, consequently, desaturation efficiency, a measure of oil frying ability. Overall, this study showed that except for the photosynthetic assimilation rate and stomatal conductance, heat, rather than drought, negatively affected the photosynthetic capacity, yield, and oil quality attributes when imposed during the flowering and silique-filling stages. This result highlights the necessity for a better understanding of heat tolerance mechanisms in crops to help to create germplasms that are adapted to rapid climate warming.

## Introduction

Alexander et al. (2006) and Yuan et al. (2016) noted the effects of anthropogenic climate change on fluctuating temperature and precipitation patterns in many biomes, including a direct effect on food security. A recent study reported that between 1964 and 2007, droughts and heat events resulted in losses of 1.82 and 1.19 billion metric tons, respectively, in cereal production (Lesk et al., 2016). Drought alone decreased the yield for wheat, rice, and maize by ~13.7% during the same period. Both the frequency and the intensity of the drought episodes and heat waves have been increasing, and the consequences for crop yields are more disastrous than those from other climatic extremes, such as flooding, frost, or hail (Pachauri et al., 2014; Zscheischler et al., 2014). The climate projections for 2100 suggest a 50% increase in the number of areas affected by drought; thus, field crop yields might decrease by more than 50% if mitigation measures are not taken (Battisti and Naylor, 2009; Li et al., 2009).

One of the mechanisms by which plants sense drought is through their roots, when the soil water potential falls below a certain threshold, and abscisic acid (ABA)-driven hormonal signaling is transduced to their leaves, triggering stomatal closure to minimize water loss by transpiration (Flexas and Medrano, 2002). This response is concomitant with a decrease in the stomatal conductance (*g*_s_) to carbon dioxide (CO_2_) uptake and the production of carbohydrates to sustain growth and development. Heat waves, which are usually synchronous with drought, exacerbate this effect by accelerating soil drying and increasing the H_2_O vapor pressure deficit (VPD) in the air, further lowering *g*_s_ (Ohsumi et al., 2008; McDowell et al., 2011). By the end of this century, the average temperature might reach as high as 3.7°C based on HadGEM2-ES derived from CMIP5 Earth System Models (ESMs) outputs, and yields could decrease from 3.1 (soybean) to 7.4% (maize) for each degree (Celsius) increase in the global mean temperature (Zhao et al., 2017). In north temperate regions of Canada, the frequency of extreme hot temperatures exceeding 30°C is expected to increase by 2080–2100 (Kharin et al., 2007). Moreover, heat stress has a specific adverse effect on crop yields if it occurs during flowering and seed development (Saini et al., 1983; Jiang et al., 2015). Pollen development, pollination, ovule fertilization, and embryo development are sensitive to heat beyond a certain temperature threshold. This is particularly true for cool-climate crops, such as canola (Prasad et al., 2017; Rieu et al., 2017).

Water availability has significant implications for carbon assimilation, a necessary source of carbohydrates for plant growth. Insufficient water results in considerable yield losses (Flexas and Medrano, 2002). In a recent meta-analysis, Yan W. et al. (2016) reported that the diffusional limitations of CO_2_ by stomata could explain 55% of the carbon assimilation decline induced by drought. Moreover, it has been shown that limitations of CO_2_ diffusion also involve resistance through the pathway from the sub-stomatal cavity to the carboxylation site of ribulose-1,5-bisphosphate carboxylase/oxygenase (RuBisCO) in the chloroplast. This component is mesophyll conductance (*g*_m_) and includes resistance by the intercellular spaces, cell walls, and membranes of the mesophyll cells and chloroplasts (Evans et al., 2009). Until recently, *g*_m_ was assumed to be steady and high enough to maintain a stable concentration of CO_2_ between the intercellular space (*C*_i_) and the chloroplast (*C*_c_) (Flexas et al., 2008). However, many studies have demonstrated that the *C*_c_ is significantly lower than the *C*_i_ and have concluded that *g*_m_ is minimal (Ethier and Livingston, 2004; Flexas et al., 2008). In addition, earlier studies reported *g*_m_ to be responsive to changes in environmental conditions, particularly heat and water deficits. This indicates a substantial limitation of photosynthetic carbon assimilation (Bernacchi et al., 2002; Warren, 2008). Under abiotic stress, the limited diffusion of CO_2_ by stomata or along the mesophyll pathway reduces the carboxylation rate (*V*_cmax_) of RuBisCO. In addition, stressors affect the enzymes involved in the catalytic reactions in the Calvin-Benson cycle and, consequently, the carboxylation rate and the regeneration rates of Ribulose 1.5-bisphoshate, also reported as the electron transport rate (*J*).

Studies on the effects of drought and heat on diffusional and biochemical limitations have reported quite different results depending on the plant species, the stress severity, and the duration and estimation methods. In addition, the specific effect of drought vs. heat on photosynthetic activity might be unclear, particularly for C_3_ crops, because of the co-occurrence of these conditions (Jagadish et al., 2011). Indeed, the respective actions of these conditions might target the different processes and structures involved in carbon assimilation to varying extents (Prasad et al., 2008). It is important, therefore, to characterize the specific effects of heat and drought on photosynthesis and yield to better understand the contribution of each of these stressors when they occur together. The identification of the traits in canola, particularly oil composition (i.e., the unsaturated vs. saturated fat ratio), affected by global warming has implications for phenotyping and breeding. In addition, these results can provide valuable information for managing irrigation when crops are exposed to heat waves and drought episodes.

The stable isotopic signatures of carbon (δ^13^C), oxygen (δ^18^O), and nitrogen (δ^15^N) in plants are influenced by environmental parameters and can be reliable indicators of plants' responses to abiotic stress, particularly a water deficit (Farquhar et al., 1982; Handley et al., 1999). At the same time, a stable isotope composition could be intrinsic and could be significantly determined by the physiological characteristics of cultivars (Yousfi et al., 2012).

*Brassica napus* L. cropped mainly for edible oil and marketed as canola, has been a major oilseed crop since the 1970s. By 2014, production had increased to 68.9 million metric tons, and the harvested area had expanded to 33.7 million hectares (FAO, 2017). Canola oil, rich in polyunsaturated fatty acids, is considered a healthy ingredient, and it is the third most used oil in foods. The meal, a by-product of oil extraction, is used for animal feed because of its high protein content (~50%), and it is ranked second in global production after soybean meal. Like other major temperate field crops, *B. napus* is particularly vulnerable to environmental stress combinations, such as heat and drought (Champolivier and Merrien, 1996; Aksouh-Harradj et al., 2006). Genetic improvement measures to mitigate this problem have provided mid- and long-term results, but the need to increase crop yields is immediate because of the growing demands for food and feed. In addition to reducing yield, heat and drought can cause deterioration in the quality of the harvested parts of crops, thereby reducing their value and profitability. Although canola is harvested for the animal feed, lubricant, and paint industries, and, recently, the bioenergy industry, edible oil remains the main reason for canola cropping. Canola oil, with its high unsaturated/saturated fatty acid ratio, provides a health benefit that is superior to that of other oilseeds. However, this attribute is subject to significant changes, such as those reported for soybean, corn, and sunflower, because of changes in the lipid biosynthesis pathways as a result of environmental factors (Martínez-Rivas et al., 2003; Baud and Lepiniec, 2010).

This study addressed the comparative effects of heat, drought, and the combination of heat and drought (hereafter referred to as heat + drought) on the carbon assimilation pathways: the main drivers of seed yield and oil content in canola. The study also sought to better understand the specific effects of stressors on the fatty acids profile because such information is determinative for oil processing and maintaining the nutritional quality. To this end, the study aimed to answer the following questions: (i) What are the effects of drought, heat, and heat + drought on the diffusional limitations of CO_2_, carboxylation and electron transport capacities? (ii) To what extent do drought and heat affect oil content and composition? Three hypotheses were offered: (i) The drought effects would prevail over the heat effects in terms of the seed yield components (the weight of the seeds, the number of siliques, and the number of seeds/silique), and the effect of each stressor would depend on the other stressor (significant interaction effect). (ii) Drought would decrease the photosynthetic assimilation rate through the stomatal and mesophyll limitations of CO_2_ diffusion, but heat would alter the photosynthetic capacity. (iii) Drought, but not heat, would decrease the oil content, but the profile of fatty acids would be altered solely by heat.

## Materials and methods

### Plant growth environment

A Canadian elite *Brassica napus* L. cultivar (N99-508), widely used in Agriculture and Agri-Food Canada's (AAFC) breeding program, was chosen for the study. To simulate field conditions, topsoil was collected from agriculture fields close to Saskatoon, Canada (52.15°N, 106.58°W), and mixed with 10% sand to provide better drainage. The soil type was chernozemic dark brown, and the texture was sandy loam with an average pH of 7.9 and 9, 23, and 295 ppm of nitrogen, phosphorus and potassium nutrients, respectively. The details of the soil characteristics are summarized in Table S1. Eight 60-liter plastic bins were filled with 50 kg of dry topsoil and slow-releasing fertilizer (Osmocote®, Everris, U.S.A.) at a rate of 10.7 g/l to avoid nutrient deficiency effects on plant growth and development. The bases and bottoms of the tubs were perforated for water drainage. In each bin, eight seeds were sown equidistantly and watered to field capacity.

The bins were divided equally between two adjacent greenhouses where the ambient day and night temperatures were 23 ± 0.5°C and 18 ± 0.5°C, respectively. The relative humidity was 45–65%. The photoperiod was set as a 16 h day and an 8 h night, and the minimum photosynthetic photon flux density (PPFD) was 400 μmol m^−2^ s^−1^ during the day. After a week of emergence, the plants were thinned, and the number was reduced to four per bin. The soil moisture was kept at field capacity by regular and light watering from emergence to bolting.

### Stress treatments

In the first greenhouse (GH-1), the daytime temperature was raised gradually and maintained at 29 ± 0.5°C from the 38th day after sowing, and the night-time temperature was maintained at 18 ± 0.5°C. Two plastic bins (8 plants) were maintained at 90% water-holding capacity (heat, H), and the remaining two plastic bins were allowed to reach 30% field capacity (heat + drought, HD) and maintained at that level until silique/pod maturation. In the second greenhouse (GH-2), the daytime temperature was maintained at 23 ± 0.5°C, and the night-time temperature was maintained at 18 ± 0.5°C, with two bins (8 plants) at 90% water-holding capacity (well-watered, WW) and the remaining bins at 30% (drought, D). From bolting to final harvest, the soil moisture and the temperature regimes were monitored in both greenhouses using a WATERMARK soil moisture monitor (Model 900M, IRROMETER CA, USA) at 1 h intervals over the experiment. The sensors were set up in two randomly assigned tubs for each treatment. The water-holding capacity of the soil was determined as follows:

$$\begin{array}{l} WHC\left(\%\right)=\;\frac{W_{sat\;+\;72}\;-\;W_{dry}}{W_{dry}}\times 100 \end{array}$$

where *W*_sat+72_ is the weight after 72 h of drainage of 20 L of water-saturated soil, and *W*_dry_ is the weight of 20 L of dry soil.

### Gas exchange measurements and isotopic discrimination

The gas exchange measurements were performed using a LI-6400XT portable photosynthesis system equipped with a 6400-08 chamber attached to a 6400-02B LED light source (LI-COR Inc., Lincoln, NE, U.S.A.) on days 9–11 after imposing the stress treatments. Measurements were made on the 4th fully developed leaf from the top (*N* = 32; four stress treatments × eight plants) between 8:30 and 11:30 a.m. The response of the net photosynthesis (*A*, μmol m^−2^ s^−1^) to the changing *C*_i_ was measured under saturated photosynthetic active radiation, PAR = 1,000 μmol m^−2^ s^−1^. The leaf was first exposed to a chamber CO_2_ concentration (*C*_a_ = 400 μmol CO_2_ mol^−1^) using CO_2_ cartridges to reach a steady state. Next, the *C*_a_ was changed in the following order: 400, 300, 200, 100, 50, 400, 500, 600, 800, 1,000 and 1,200 μmol mol^−1^. This was done while ensuring that the net photosynthetic assimilation rate (*A*), water vapor, and CO_2_ fractions reached steady values at each step before moving to the next. During the measurement periods, the leaf chamber temperatures were kept at 23°C and 29°C depending on the greenhouse conditions: air flow at 500 μmol s^−1^, relative humidity at 55–65%, and VPD at 1.2 ± 0.1 KPa. The order of the measurements was randomized among the treatments and the days and along the measuring period. The *A* (μmol CO_2_ m^−2^ s^−1^) and the *g*_s_ (mol CO_2_ m^−2^ s^−1^) values were extracted from the *A*–*C*_i_ response measurements for *C*_a_ = 400 μmol CO_2_ mol^−1^ (atmospheric ambient CO_2_ concentration)_._ The intrinsic water-use efficiency (WUE_i_) was then deduced (WUE_i_ = *A*/*g*_s_).

The maximum rate of RuBisCO carboxylation (*V*_cmax_, μmol m^−2^ s^−1^), the rate of photochemical electron transport (*J*, μmol e^−^ m^−2^ s^−1^), and the rate of CO_2_ diffusion from the *C*_i_ to the *C*_c_ carboxylation site or *g*_m_ (*g*_m_, mol m^−2^ s^−1^) were estimated by *A*-*C*_i_ curve fitting, according to Ethier and Livingston (2004) and Ethier et al. (2006), with the biochemical model of C_3_ photosynthesis developed by Farquhar et al. (1980).

The final rate, *A*, was:

$$\begin{array}{l} A=min\;\left(Ac,Aj\right) \end{array}$$

The RuBisCO-limited rate of CO_2_ assimilation (*A*_*c*_) was given by:

$$\begin{array}{l} A_{c}=\;\frac{\left(C_{i}-\Gamma^{*}\right)V_{cmax}}{C_{i}+K_{c}\left(1\;+O/K_{o}\right)}-R_{d} \end{array}$$

where *V*_cmax_ is the maximum rate of carboxylation, *C*_c_ is the chloroplast concentration of CO_2_, *C*_i_ is the intercellular concentration of CO_2_, Γ^*^ is the CO_2_ compensation point, and *K*_c_ and *K*_o_, are the Michaelis–Menten constants of RuBisCO for CO_2_ and O_2_, respectively.

The RuBP-limited rate of CO_2_ assimilation (*A*_*j*_) was given by:

$$\begin{array}{l} A_{j}\;=\;\frac{\left(C_{i}-\Gamma^{*}\right)J/4}{C_{i}\;+\;2\Gamma^{*}}-R_{d} \end{array}$$

where *J* is the rate of electron transport. The rate of electron transport was given by:

$$\begin{array}{l} J=\frac{4\left(A_{j}+R_{d}\right)\left(C_{i}-\frac{A_{j}}{g_{i}}\;+\;2\Gamma^{*}\right)}{\left(C_{i}-\frac{A_{j}}{g_{i}}-\Gamma^{*}\right)} \end{array}$$

The *g*_m_ was calculated from:

$$\begin{array}{l} {\frac{1}{g}}_{m}={\frac{1}{g}}_{i}\;+\;{\frac{1}{K}}_{c} \end{array}$$

where

$$\begin{array}{l} g_{i}\;=\;\frac{A}{C_{i}-C_{c}} \end{array}$$

and *K*_c_ is the carboxylation efficiency (the initial slope of the *A-C*_c_ curve)

$$\begin{array}{l} K_{c}=\frac{dA}{dC_{c}} \end{array}$$

Once the gas exchange measurements were recorded from the very same 4th leaf, 10 leaf punches (5 mm diameter) were collected, using a single-hole punch, on either side of the midrib toward the stable isotopic composition of carbon (δ^13^C), nitrogen (δ^15^N), and oxygen (δ^18^O), as well as the carbon:nitrogen (C:N) ratio. Later, the leaf punches were oven-dried at 60°C for 72 h at constant mass. Four (4) of the leaf discs were weighed and individually packed in tin (δ^13^C and δ^15^N) and silver (δ^18^O) capsules. They were then sent to the University of California at Davis Stable Isotope Facility to be combusted and analyzed by an online continuous flow dual analyzer coupled to an isotope ratio mass spectrometer (Europa Scientific Integra, Cheshire, England, UK). The values for leaf discrimination against the heavy isotope Δ^13^C were calculated, following Farquhar et al. (1989), as:

$$\begin{array}{l} \Delta^{13}C=\frac{\delta^{13}C_{a}-\;\delta^{13}C_{leaf}}{1\;+\;\delta^{13}C_{leaf}} \end{array}$$

where δ^13^C_a_ and δ^13^C_leaf_ are, respectively, the carbon isotope composition of the ambient air (−8.3%0) and the leaf sample with, as reference, the Vienna PeeDee Belemnite (VPDB) carbonate standard, according to the following formula:

$$\delta^{13}C=\frac{{{(}^{13}C{/}^{12}C)}_{sample}-\;{{(}^{13}C{/}^{12}C)}_{VPDB}}{{{(}^{13}C{/}^{12}C)}_{VPDB}\cdot 1,000}$$

where (^13^C/^12^C)_sample_ and (^13^C/^12^C)_VPDB_ are the ratios of ^13^C and ^12^C in the sample and the VPDB, respectively.

In the same way, δ^15^N and δ^18^O were calculated as:

$$\begin{array}{l} \;\delta^{15}N=\frac{{{(}^{15}N{/}^{14}N)}_{sample}-{{(}^{15}N{/}^{14}N)}_{AIR}}{{{(}^{15}N{/}^{14}N)}_{AIR}\cdot 1,000}\\ \delta^{18}O=\frac{{{(}^{18}O{/}^{16}O)}_{sample}-\;{{(}^{18}O{/}^{16}O)}_{VSMOW}}{{{(}^{18}O{/}^{16}O)}_{VSMOW}\cdot 1,000} \end{array}$$

The Vienna standard mean ocean water (VSMOW) and atmospheric nitrogen were the standards used to calculate the δ^18^O and δ^15^N, respectively. All of the isotopic values were expressed in per mil (%0), and the error of the repeated measurements did not exceed 0.1%0.

### Abscisic acid content

A fully expanded leaf (5th leaf from the top) was sampled and immediately packed in Eppendorf tubes and frozen in liquid nitrogen before being stored in a −80°C freezer until processed for analysis. The ABA content was determined as described in Yan D. et al. (2016). The samples were centrifuged to remove debris, and the pellet was washed twice. The supernatant was evaporated in a SpeedVac, reconstituted in 1 ml of 1% (v/v) acetic acid, and purified by solid phase extraction using Oasis HLB, MCX, and WAX cartridge columns (Waters Limited, Mississauga ON, Canada). The solvent was removed under vacuum and subjected to the LC-ESI-MS/MS analysis (Agilent 6410 TripleQuad LC/MS system). A Liquid Chromatography (Agilent 1200 series) equipped with a 50 × 2.1 mm, 1.8 μm Zorbax SB-Phenyl column (Agilent) was used with a binary solvent system comprising 0.01% (v/v) acetic acid in water (Solvent A) and 0.05% (v/v) acetic acid in acetonitrile (Solvent B). The separations were performed using a gradient of increasing acetonitrile content with a flow rate of 0.2 ml min^−1^. The gradient was increased linearly from 3 to 50% B over 15 min. The retention time for the ABA was 14.0 min.

### Growth

The normalized difference vegetation index (NDVI) was measured using a GreenSeeker handheld crop sensor (Trimble, Westminster CO, USA). The sensor was held 80 cm above the plant canopy, as recommended by the manufacturer. The measurements were taken between 9 and 11 a.m. just before flowering started. Plant height was measured at physiological maturity.

### Harvest

Upon reaching physiological maturity, the siliques were harvested, counted, and stored in a dryer at 23°C for 4–5 days before threshing. The seeds were then collected, counted, and weighed. Later, the seeds were sent to AAFC's oil chemistry lab in Saskatoon for oil and protein analyses. To qualify the effects of the combined vs. the single stressors on the yield traits and the photosynthetic *A*, the effect weights of drought (D), heat (H), and heat + drought (HD), compared to the well-watered (WW) treatment were calculated using the following formula:

$$\begin{array}{l} T_{e}=\;\frac{X_{t}-{\bar{X}}_{WW}}{{\bar{X}}_{WW}} \end{array}$$

where *T*_*e*_ is the treatment effect weight, *X*_*t*_ is the trait “*X*” value for the treatment *T*, and ${\bar{X}}_{WW}$ is the corresponding mean value for the well-watered plants. The heat + drought effect weight obtained with the above formula (HD_e_) was compared to the calculated heat + drought effect (*HD*_calc_) using the following formula:

$$\begin{array}{l} HD_{calc}={\bar{H}}_{e}+{\bar{D}}_{e}-{\bar{H}}_{e}\times {\bar{D}}_{e} \end{array}$$

where ${\bar{H}}_{e}$ and ${\bar{D}}_{e}$ are the means of the heat and drought effect weights, respectively (Darling et al., 2010; Bansal et al., 2013).

### Fatty acid and protein content of seeds

The seeds were pooled from each stress treatment and further divided into three sub-samples for analyses of the total oil content, fatty acid composition, and total protein content. The seed oil fatty acyl composition was analyzed using gas chromatography (GC) following the preparation of the fatty acid methyl esters by base-catalyzed methanolysis (Thies, 1971) and according to the protocol detailed in Heydarian et al. (2016). The individual fatty acids were reported as a percentage of the total fatty acid methyl esters by mass. The total oil content was calculated as the sum of the content of the individual triglycerides. The seed protein content was determined by the American Oil Chemists' Society's generic combustion method for crude protein (Official Method Ba 4e-93). Combustion at a high temperature in pure oxygen frees nitrogen, which is measured by thermal conductivity detection and then converted to the equivalent protein by an appropriate numerical factor (AOAC, 2003). A LECO FP-528 protein analyzer was used, and the results were reported as a percentage, *N* × 6.25, calculated on a whole-seed dry matter (zero moisture) basis. Subsequently, the ω-3 desaturation efficiency (DE) and the ω-6 DE were deduced from the profile of the fatty acids and calculated according to Menard et al. (2017):

$$\begin{array}{l} \omega -3\;DE=\frac{18\;:\;3}{18\;:\;2\;+\;18\;:\;3}\\ \omega -6\;DE=\frac{18\;:\;2\;+\;18\;:\;3}{18\;:\;1\;+\;18\;:\;2\;+\;18\;:\;3} \end{array}$$

### Statistical analyses

A two-way analysis of variance (ANOVA) was used to test the effects of the ambient temperature, the water status, and the interaction between them on the measured traits. The means were compared using Tukey's honest significant difference (HSD) at a *p* < 0.05 significance level. The coefficients and the *p*-values of the correlations were calculated using Pearson's correlation coefficient. All of the statistical analyses were performed with R software version 3.2.2 (R Core Team, 2015).

## Results

Heat had a significant effect on the photosynthetic-related variables (*A, V*_cmax_, *J*, and *g*_m_) but not on *g*_s_, WUE_i_ and Δ^13^C (Table 1). Heat also affected the growth attributes (plant height and NDVI) and all of the measured yield and oil quality attributes. In addition, the δ^15^N levels were significantly affected by heat alone. The available soil moisture status (drought) had a significant effect on all of the photosynthesis variables except *g*_m_, the growth and yield attributes, and the ABA and seed protein content (Table 1). The heat + drought interaction did not significantly affect the measured variables except for the Δ^13^C and the oil content (Table 1). It had a marginally significant effect (*p* = 0.06) on the NDVI and the seed weight.

**Table 1:** ANOVA of photosynthetic capacity attributes, yield, and seed composition of an elite canola cultivar in response to drought, heat, and heat + drought.

	***A***	***g*_s_**	**WUE_i_**	***V*_cmax_**	**J**	***g*_m_**	**C:N**	**Δ^13^C**	**δ^15^N**	**δ^18^O**	**Ht**	**NDVI**	**ABA**	**Silique number**	**Seed weight**	**Seed number**	**Oil content (% DM)**	**ω-3 DE**	**ω-6 DE**	**Seed protein**
**SOURCE OF VARIATION**																				
H	***	ns	ns	***	***	***	ns	ns	***	ns	**	***	ns	***	***	***	***	**	**	***
D	***	***	***	***	***	ns	***	***	ns	ns	**	***	***	**	*	***	***	ns	ns	***
H × D	ns	ns	ns	ns	ns	ns	ns	***	ns	ns	ns	ns^±^	ns	ns	ns^±^	ns	***	ns	ns	ns

A, Net photosynthetic assimilation rate (μmol CO_2_ m^−2^ s^−1^); g_s_, stomatal conductance (mol CO_2_ m^−2^ s^−1^); WUE_i_, intrinsic water-use efficiency (μmol CO_2_ mol^−1^ H_2_O); V_cmax_, maximal carboxylation rate of CO_2_ (μmol CO_2_ m^−2^ s^−1^); J, photosynthetic electron transport rate (μmol CO_2_ m^−2^ s^−1^); g_m_, mesophyll conductance (mol CO_2_ m^−2^ s^−1^); C:N, carbon to nitrogen ratio; Δ^13^C, isotopic composition of leaf carbon (%0); δ^15^N, isotopic composition of leaf nitrogen (%0); δ^18^O, isotopic composition of leaf oxygen (%0); Ht, plant height (cm); NDVI, normalized difference vegetation index; ABA, abscisic acid leaf content (ng g^−1^ of dry weight); ω-3 DE, desaturation efficiency of ω-3 fatty acids; ω-6 DE, desaturation efficiency of ω-6 fatty acids. ***p < 0.001; **0.001 ≤ p < 0.01; *0.01 ≤ p < 0.05; ns, non-significant; ns^±^, marginally significant (p = 0.06).

### Photosynthetic carbon fixation capacity and growth

**Figure 1:**
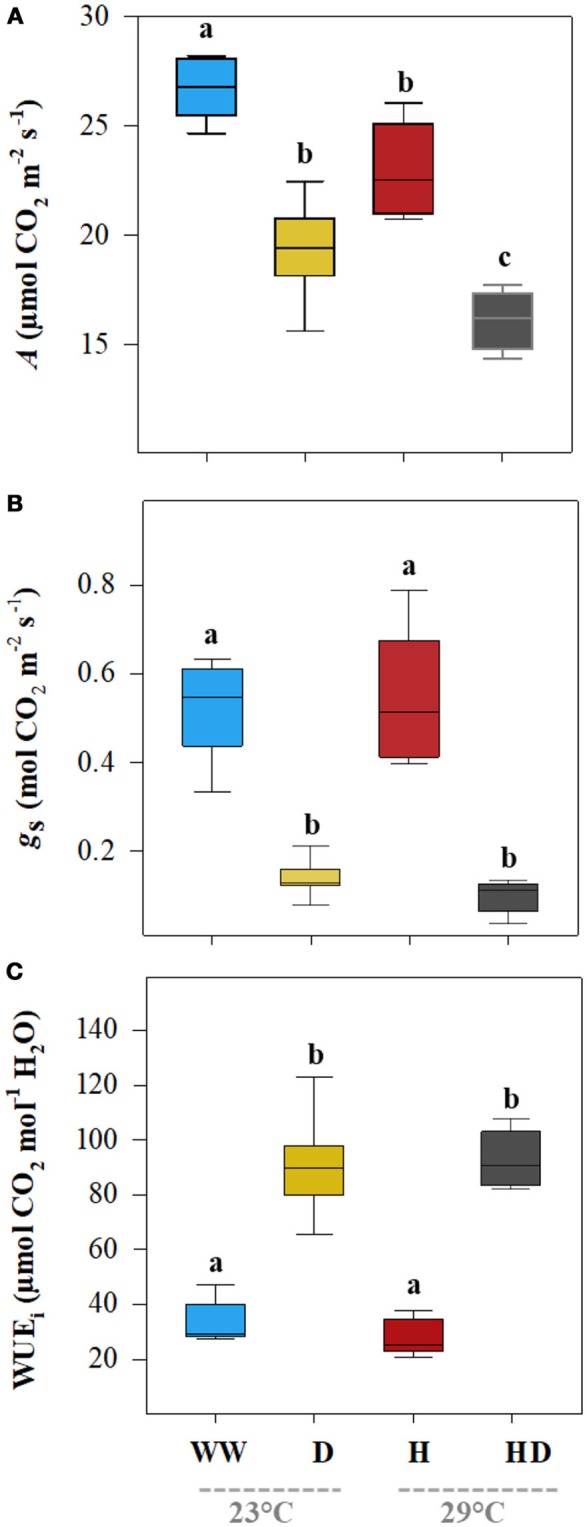
The leaf net photosynthetic assimilation rate (*A*, **A**), stomatal conductance of CO_2_ (*g*_s_, **B**), and intrinsic water use efficiency (WUE_*i*_ = *A/g*_s_, **C**) of the plants grown under the well-watered (WW), drought (D), heat (H), and heat + drought (HD) treatments. The statistically significant differences among the treatments are labeled with different letters at *p* < 0.05 (Tukey's HSD). The box ends indicate the upper (3rd) to lower (1st) quartiles of the value ranges, and the whiskers indicate the highest and lowest observations. The horizontal line inside the box marks the median for the observations.

**Figure 2:**
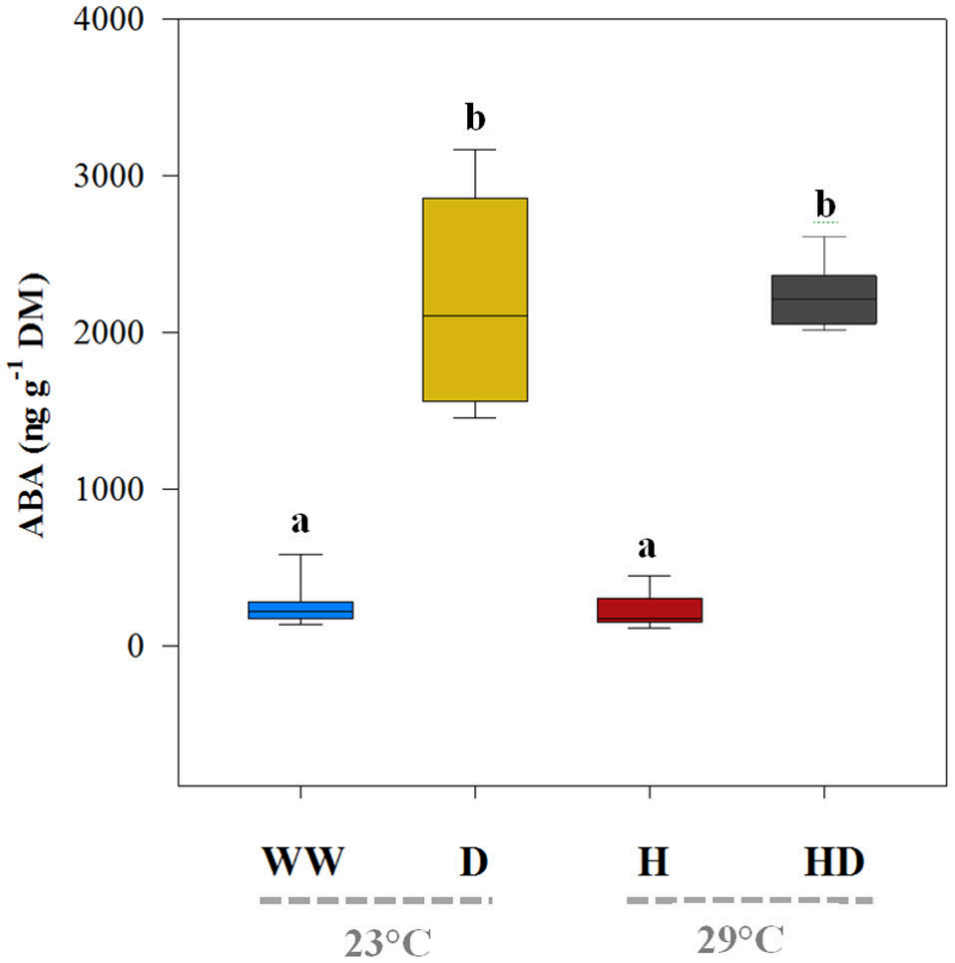
The leaf abscisic acid content (ABA) of the plants exposed to the well-watered (WW), drought (D), heat (H), and heat + drought (HD) treatments. The statistically significant differences among the treatments are labeled with different letters at *p* < 0.05 (Tukey's HSD). The box ends indicate the upper (3rd) to lower (1st) quartiles of the value ranges, and the whiskers indicate the highest and lowest observations. The horizontal line inside the box marks the median for the observations.

Although heat and drought significantly reduced the net photosynthetic *A*, the heat + drought treatment had the greatest effect: the *A* was ~55% less than the *A* for the well-watered plants (Table 2). In addition, the drought treatment had a greater effect on the *A* (19.4 μmol CO_2_ m^−2^ s^−1^) than did the heat treatment (22.45 μmol CO_2_ m^−2^ s^−1^); however, the well-watered plants maintained the highest photosynthetic carbon fixation capacity (26.74 μmol CO_2_ m^−2^ s^−1^; see Figure 1A). Compared to the *g*_s_ under the well-watered treatment (0.52 mol CO_2_ m^−2^ s^−1^), the *g*_s_ for the plants exposed to the heat treatment did not change significantly (0.48 mol CO_2_ m^−2^ s^−1^); however, it dropped sharply in the plants exposed to drought (0.14 mol CO_2_ m^−2^ s^−1^; see Figure 1B). The WUE_i_ was similar between the treatments (well-watered and heat) when water was supplied, averaging 30.75 μmol CO_2_ mol^−1^ H_2_O (Figure 1C). In contrast, the WUE_i_ rose significantly, by ~173%, in the plants subjected to a water deficit (drought and heat + drought treatments). The leaf ABA content, being low under the well-watered and heat treatments (219.1 and 198.1 ng g^−1^ DM, respectively), mirrored the WUE_i_ patterns (Figure 2) and increased noticeably when the plants were exposed to a soil water deficit under drought and heat + drought (2,195 and 2,209 ng g^−1^ DM, respectively).

**Table 2:** Effect weight of net photosynthetic assimilation rate (*A*) and yield traits as the deviation of the trait value under single and combined stressors on the value under the control conditions (well-watered plants).

	**D_e_**	**H_e_**	**HD_e_**	**H + D_calc_**	**Result of combined stressors**
*A*	0.28	0.16	0.55	0.39	Synergistic
Silique number	0.49	0.59	0.82	0.80	Cumulative
Seed weight	0.31	0.85	0.89	0.90	Heat dominant
Seeds number	0.36	0.70	0.85	0.79	Cumulative
Oil content (%DM)	Null	0.53	0.19	0.53	Antagonistic

*D_e_, effect weight of drought treatment (vs. well-watered plants); H_e_, effect weight of drought treatment; HD_e_, effect weight of heat and drought combination; H + D_calc_, calculated effect weight of heat and drought combination*.

**Figure 3:**
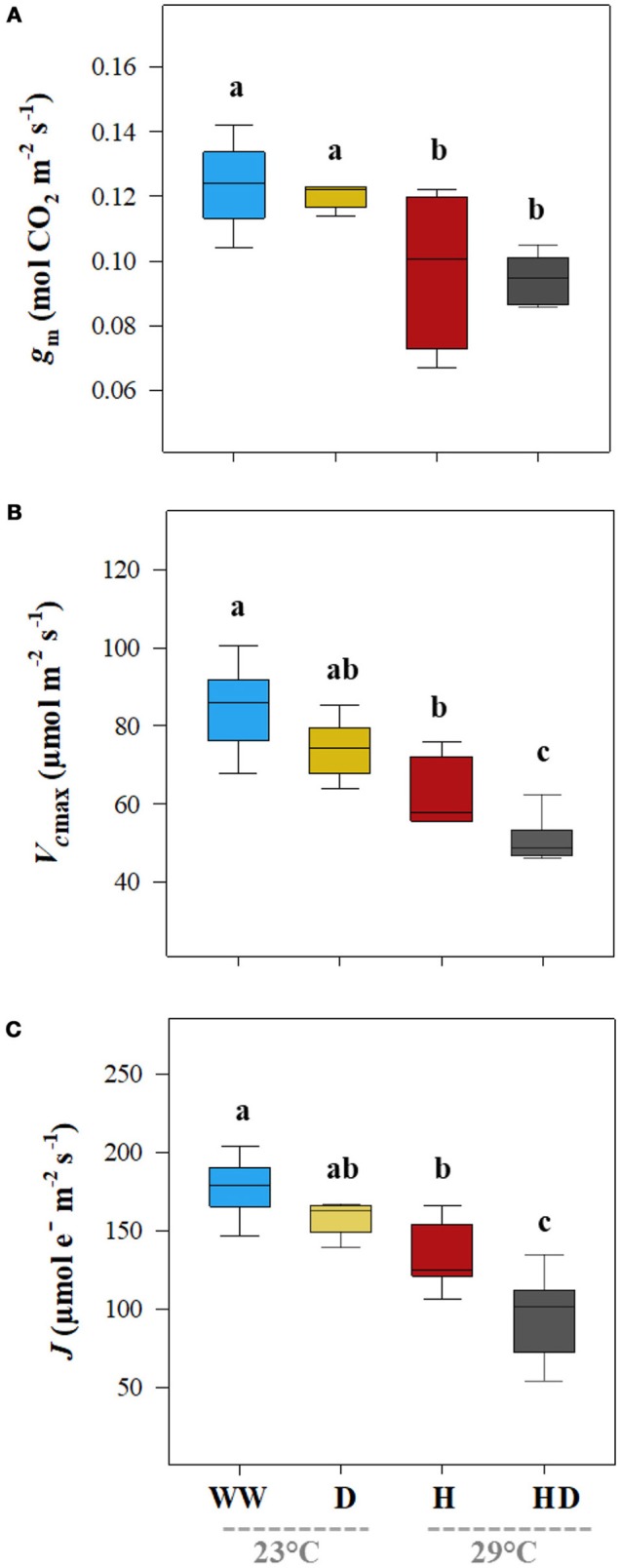
The leaf mesophyll conductance for CO_2_ (*g*_m_, **A**), the maximum carboxylation rate of ribulose-1,5-bisphosphate carboxylase/oxygenase (*Vc*_max_, **B**), and the photosynthetic rate of the electron transport (*J*, **C**) for the plants exposed to the well-watered (WW), drought (D), heat (H), and heat +drought (HD) treatments. The statistically significant differences among the treatments are labeled with different letters at *p* < 0.05 (Tukey's HSD). The box ends indicate the upper (3rd) to lower (1st) quartiles of the value ranges, and the whiskers indicate the highest and lowest observations. The horizontal line inside the box marks the median for the observations.

Unlike the result for the *g*_s_, the *g*_m_ decreased significantly on exposure to heat (25%) but did not change under the drought treatment (0.13 mol CO_2_ m^−2^ s^−1^; see Figure 3A). The heat + drought combination decreased *g*_s_ and *g*_m_ significantly; however, the effect of the single stressors was not significantly greater. Compared to those for the well-watered plants, the maximal *V*_cmax_ and *J* were reduced under the heat + drought treatment and, to a lesser extent, under the heat but not the drought treatment, following a similar trend as that for *g*_m_ (Figures 3B,C). The *V*_cmax_ was 84.4 μmol m^−2^ s^−1^ under the well-watered treatment and dropped to 63.2 and 43.9 μmol m^−2^ s^−1^ in the plants exposed to the heat and heat + drought treatments, respectively (Figure 3B). The *J* followed a similar pattern as that for the *V*_cmax_, going from 178.2 μmol e^−^ m^−2^ s^−1^ for the well-watered plants to 133.2 and 95.4 μmol e^−^ m^−2^ s^−1^ for the plants exposed to the heat and heat + drought treatments, respectively (Figure 3C). Plant height averaged 145.8 cm under the control conditions and decreased under all the stress treatments. Height was the most affected by the heat + drought treatment, decreasing by 35.8% as compared to the well-watered plants (data not shown). The NDVI was 0.76 for the well-watered plants. It decreased under the drought and heat + drought treatments to 0.69 and 0.60, respectively. However, the NDVI was not affected by the heat treatment.

### Resource-use efficiencies

**Figure 4:**
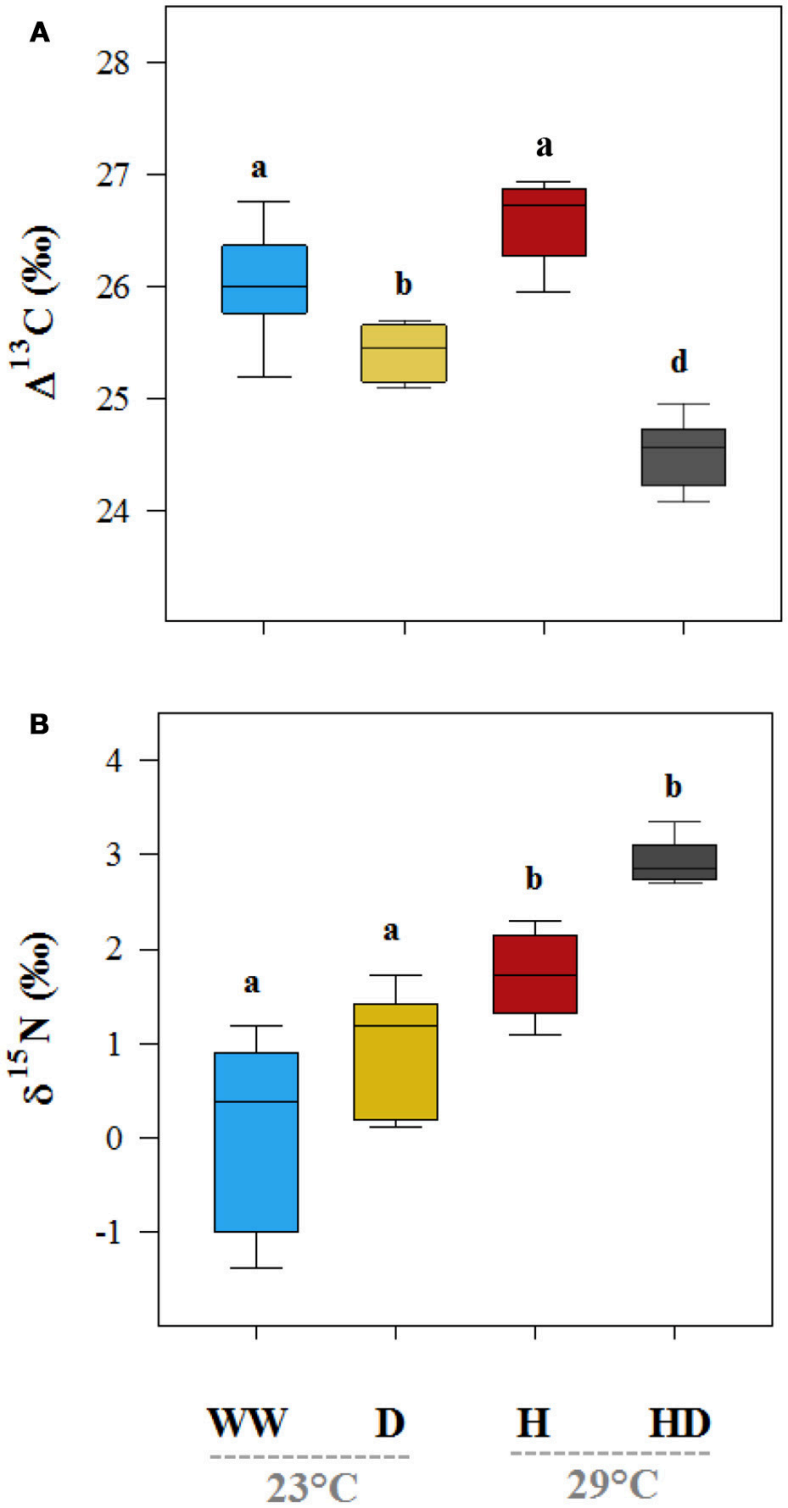
The effects of the well-watered (WW), drought (D), heat (H), and heat + drought (HD) treatments on leaf carbon (Δ^13^C, **A**) and the nitrogen (δ^15^N, **B**) isotopic composition. Different letters denote significantly different treatments at *p* < 0.05 (Tukey's HSD). The box ends indicate the upper (3rd) to lower (1st) quartiles of the value ranges, and the whiskers indicate the highest and lowest observations. The horizontal line inside the box marks the median for the observations.

**Figure 5:**
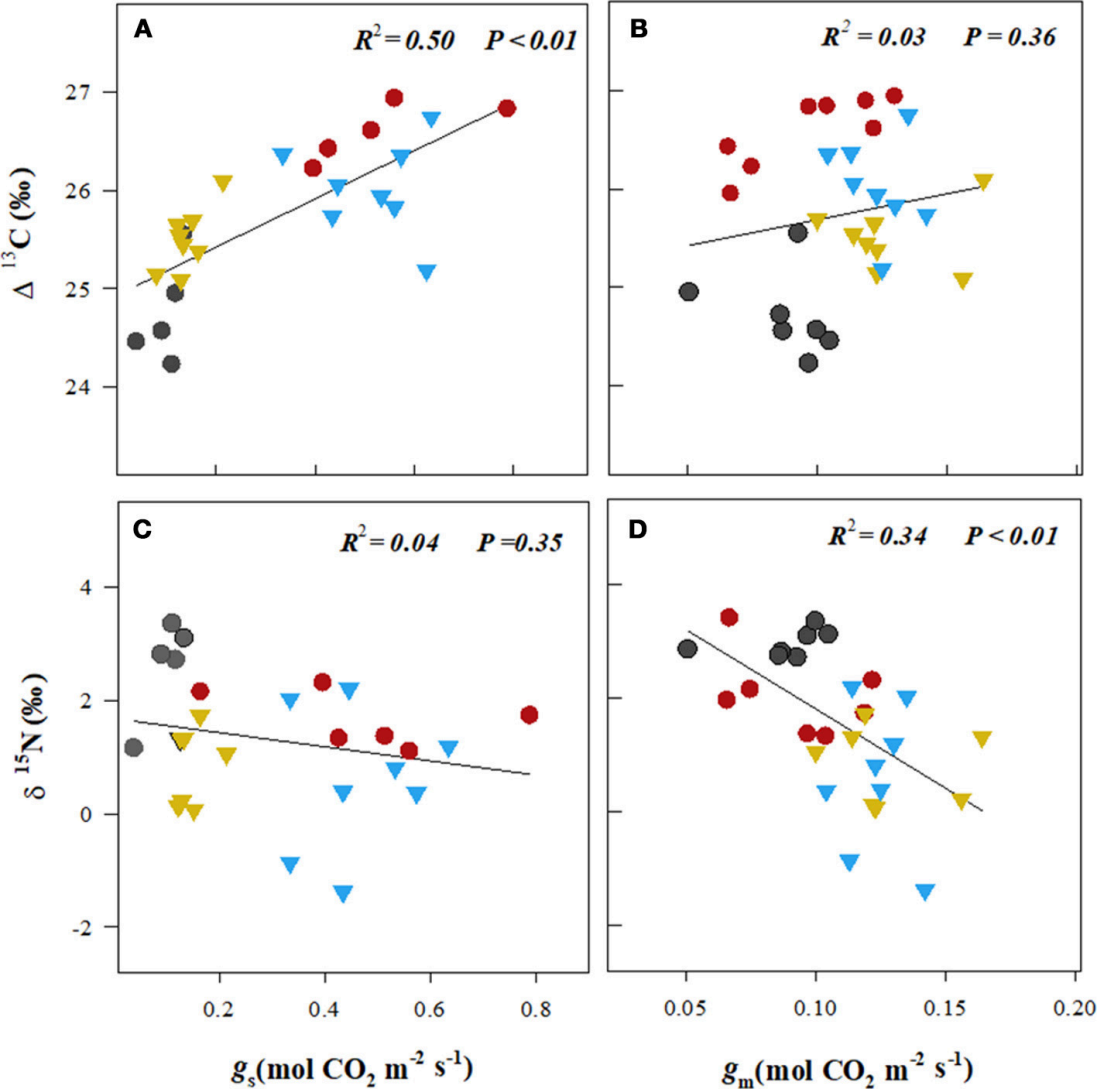
The relationships between leaf stomatal conductance (*g*_s_, **A,C**) and mesophyll conductance (*g*_m_, **B,D**) of CO_2_ and the leaf isotopic composition of carbon (Δ^13^C) and nitrogen (δ^15^N). 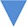, well-watered plants (WW, 23°C); 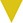, drought (D, 23°C); 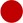, heat (H, 29°C); 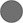, heat + drought (HD, 29°C). The lines were fitted by regression using all the points in a plot.

The isotopic composition of the leaf carbon, Δ^13^C (%0), was sensitive to the stress treatments, and discrimination reduced on exposure to heat + drought and drought (Figure 4A). The observed pattern in the Δ^13^C was similar to that in the *g*_s_, where a significant correlation was found between *g*_s_ and the Δ^13^C (*R*^2^ = 0.50, *p* < 0.01; see Figure 5A), but no correlation was found between the *g*_m_ and the Δ^13^C (*R*^2^ = 0.03, *p* = 0.36; see Figure 5B). This result suggests that carbon discrimination is driven mainly by stomatal closure. The treatment differences in the δ^15^N were more noticeable, and the values ranged from 0.59%0 (well-watered) to 2.72%0 (heat + drought; see Figure 4B). The δ^15^N plotted against the *g*_m_ showed a significant negative correlation (*R*^2^ = 0.34, *p* < 0.01; see Figure 5D), while no significant correlation was found between δ^15^N and *g*_*s*_ (Figure 5C). Similarly, both the *V*_cmax_ and the *J* were negatively correlated with the δ^15^N (*R*^2^ = 0.32 and 0.27, *p* < 0.01, respectively; see Figure S1). The observed δ^18^O was not significantly different for the treatments, and the average value was 17.82%0 (Table 1).

### Seed yield and total oil and protein content

**Figure 6:**
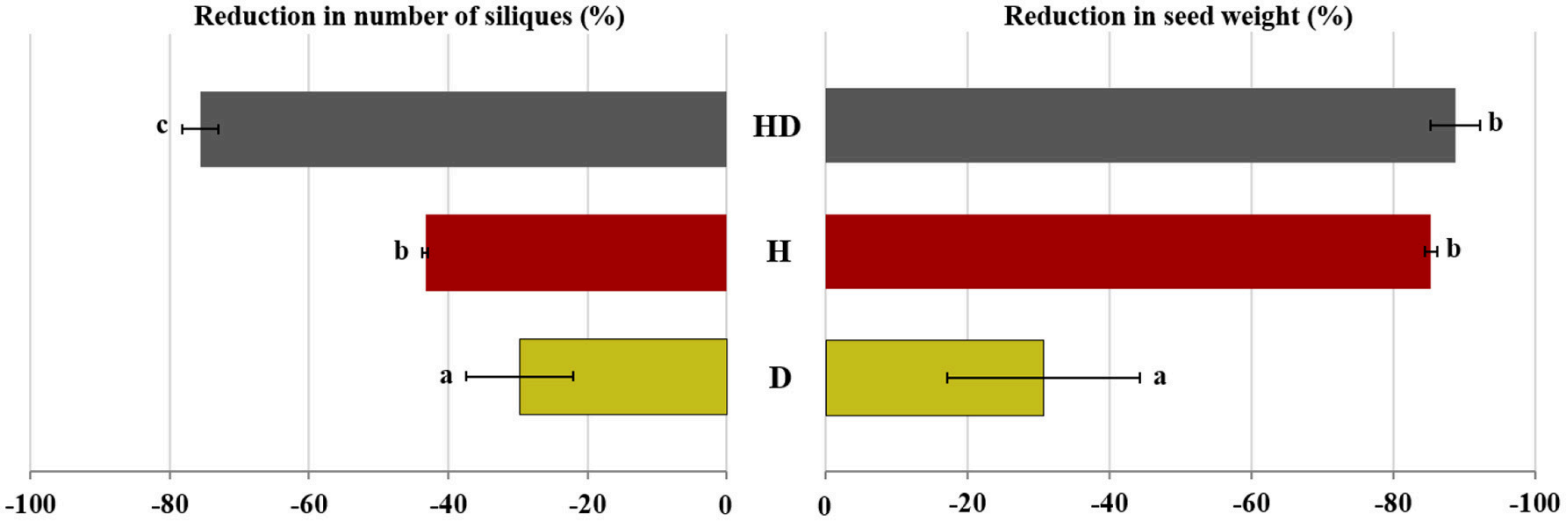
The percentage reduction in the seed weight and number of silique for the plants subjected to the drought (D), heat (H), and heat + drought (HD) treatments compared to the plants subjected to the control treatment. The treatments that were significantly different at *p* < 0.05 are labeled with different letters (Tukey's HSD).

The number of siliques decreased noticeably by exposure to heat + drought (76%) and less by heat (43%) compared to the well-watered plants, which had 178 siliques/plant on average (Figure 6). The plants exposed to drought were less affected, with 125 siliques per plant. The seed yield was highest for the well-watered plants (6.46 g/plant). It diminished by 85% and 89% for the plants exposed to heat and heat + drought, respectively, and by 31% for the plants subjected to drought (Table 2).

**Figure 7:**
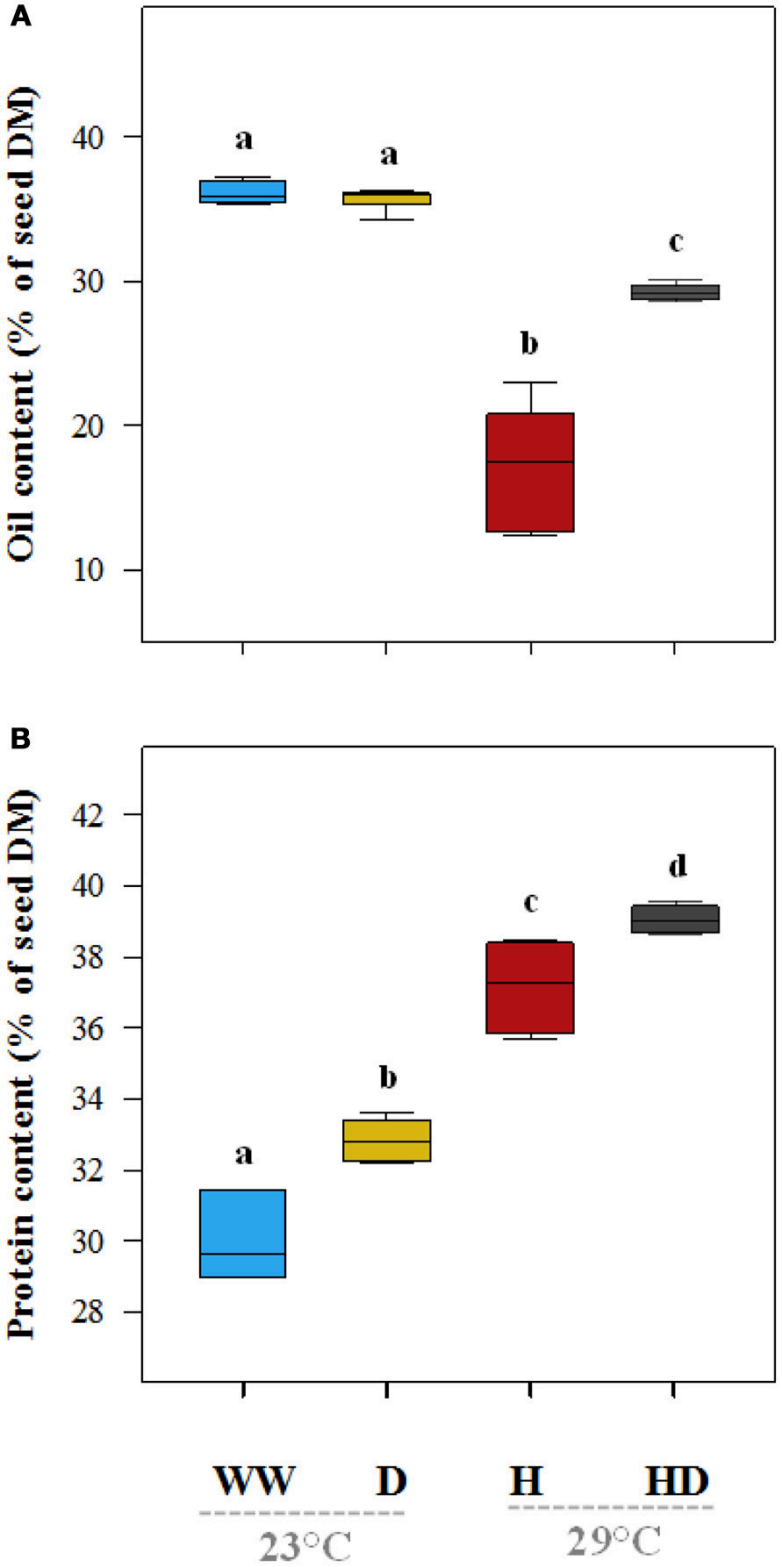
The total oil **(A)** and total protein **(B)** content of the seeds under the different treatments expressed as a percentage of dry matter (% DM). The treatments that were significantly different at *p* < 0.05 are labeled with different letters (Tukey's HSD). The box ends indicate the upper (3rd) to lower (1st) quartiles of the value ranges, and the whiskers indicate the highest and lowest observations. The horizontal line inside the box marks the median for the observations.

**Figure 8:**
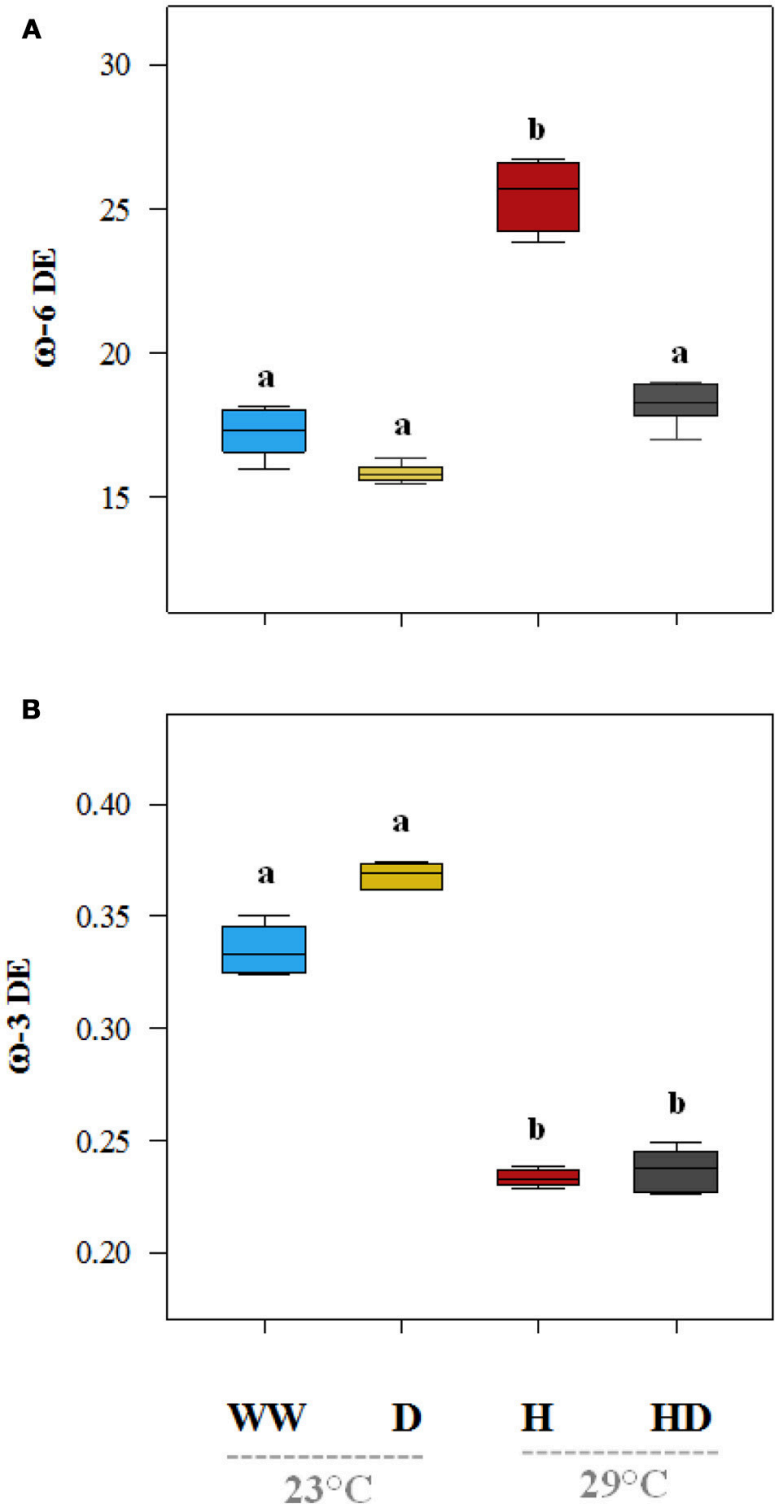
The desaturation efficiency (DE) of the Omega 6 (ω-6) **(A)** and Omega 3 (ω-3) **(B)** of the seed fatty acids under the different treatments. The DE values that were significantly different at *p* < 0.05 are labeled with different letters (Tukey's HSD). The box ends indicate the upper (3rd) to lower (1st) quartiles of the value ranges, and the whiskers indicate the highest and lowest observations. The horizontal line inside the box marks the median for the observations.

On average, the oil content (% of seed dry matter) of the plants under the heat treatment was particularly low (17.2%) compared to 36.1% in the seeds of the well-watered plants (Figure 7A). The oil content (35.7%) was not significantly affected by drought, but when heat and drought were combined, it dropped to 29.2%. The total protein content (% dry matter) was 30% in the seeds of the well-watered plants. It increased under all of the stress treatments (drought 32.8%, heat 37.2%, and heat + drought 39%; see Figure 7B). The DE of the ω-6 fatty acids increased significantly as compared to the DE in the well-watered plants (17.3) when the plants were exposed to the heat treatment (25.5), but it did not change significantly when the plants were exposed to the drought and the heat + drought treatments (Figure 8A). The drought and heat + drought stress treatments increased the unsaturated fatty acid fraction. The heat treatment increased the saturated fatty acid fraction, but the drought treatment lowered it (Figure S2). The heat application resulted in a decrease in the oleic acid (18:1) content and an increase in the linoleic acid (18:2; see Figure S2). In contrast, the plants exposed to heat and heat + drought had a lower ω-3 DE (0.24 on average) than the well-watered plants (0.33; see Figure 8B) as the α-Linolenic acid (18:3) content decreased and the linoleic (18:2) increased (Figure S2).

### Relationships among photosynthesis, resource-use efficiency, and yield

**Figure 9:**
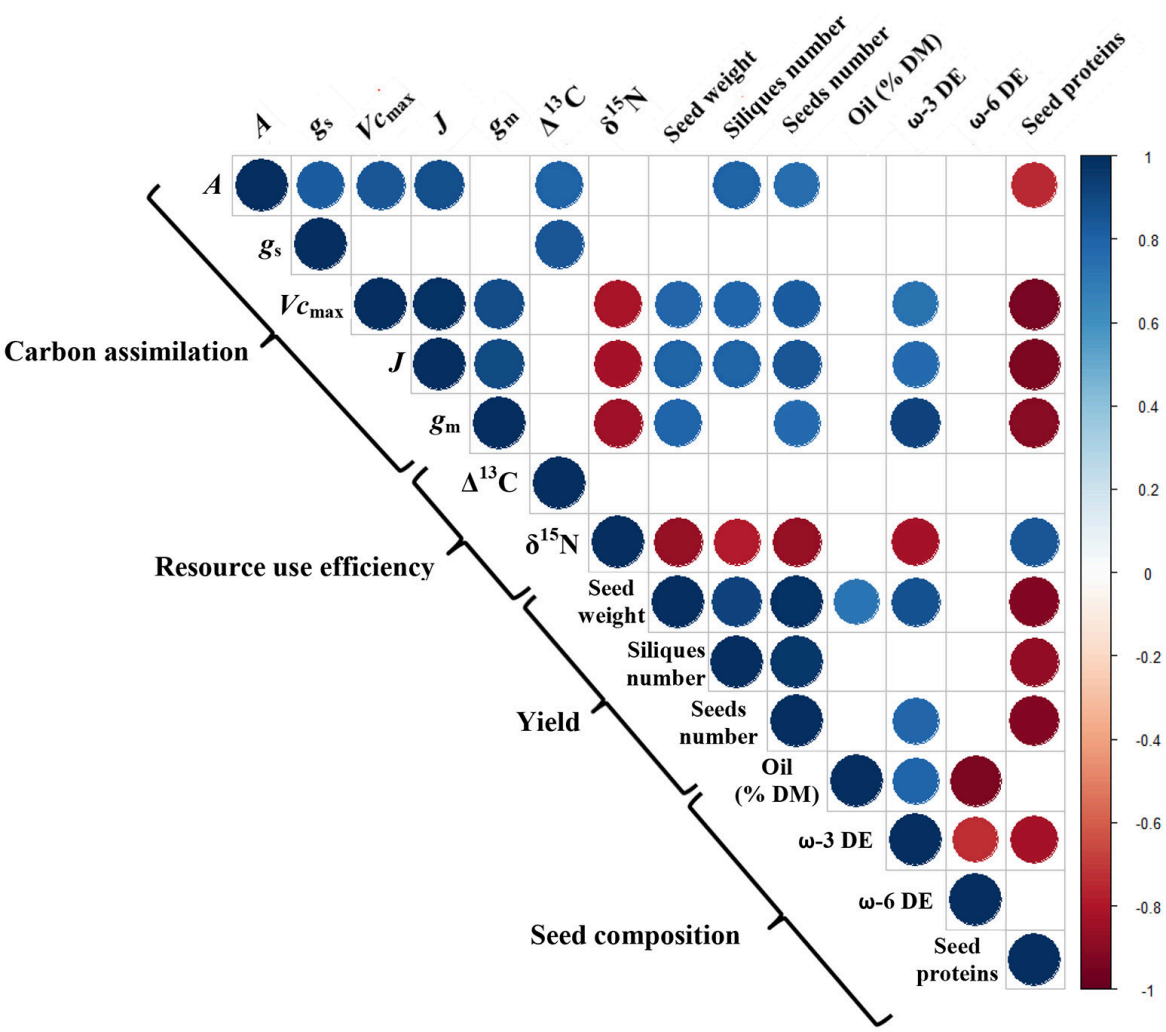
The Pearson's correlation coefficients for the measured traits across all the treatments. The blue and red circles denote significant positive and negative correlations, respectively (*p* = 0.05), and the empty cases refer to non-significant correlations. The color gradient is proportional to the correlation coefficient.

The biochemical limitations of photosynthesis (*V*_cmax_, *J*, and *g*_m_) and yield attributes (silique number, seed number, and seed weight) were negatively correlated with the δ^15^N, but a positive correlation was observed between the δ^15^N and the seed protein content (Figure 9). As for the seed composition, the oil content was positively correlated with the physiological variables (*A, V*_*c*max_, *J*, and *g*_m_) and with the ω-3 DE (*R*^2^ = 0.63) but negatively related to the ω-6 DE (*R*^2^ = 0.88).

## Discussion

The results of this work emphasizes an exacerbating effect of combined heat and drought on spring canola growth and yield, compared to single applications of stressors as reported for other crops (Nankishore and Farrell, 2016; Mahrookashani et al., 2017; Sehgal et al., 2017). In addition, we noticed a prevailing effect of heat over drought on photosynthetic capacity, yield and seed composition traits, most likely due to the deleterious effect of heat on the enzymes involved in carbon assimilation and metabolism. However, drought specifically affected stomatal conductance of CO_2_ and related traits (ABA content, Δ^13^C and net assimilation rate).

### Response of CO_2_ diffusion and photosynthetic capacity to heat and drought

Photosynthetic activity is sensitive to both drought and heat, particularly for C_3_ metabolic pathway crops, and the degree of tolerance to stressors is determinant for their survival (Feller and Vaseva, 2014). The rapid acclimation of photosynthesis to abiotic stressors has been reported for many species, particularly in response to drought and, to a lesser extent, heat. Acclimation, also termed phenotypic plasticity, consists of adjustments of physiological traits, resulting in a limited decline in growth performance (Sadras et al., 2009).

In this experiment, *g*_s_ was affected when plants were subjected to a water deficit (drought and heat + drought treatments). Under the drought conditions and the optimal temperature (23°C), the ABA leaf content increased dramatically leading to stomatal closure. This suggests that the ABA signaling pathway triggered stomatal closure to reduce the loss of tissue turgor (Wilkinson and Davies, 2010; Pantin et al., 2013). However, keeping the stomata open at a high temperature and in increased leaf-to-air VPD conditions ensures leaf transpirational cooling, but this is conditional to soil moisture availability in the root zone (Crawford et al., 2012). The findings of this study are consistent with those of previous studies. High *g*_s_ was seen in plants subjected to the heat (29°C) and the well-watered (23°C) conditions, and low *g*_s_ was observed under the drought and the heat + drought conditions. Overall, the response of the canola plants seemed to be a “conservational” strategy driven by water economy via stomatal closing rather than leaf cooling. This resulted in greater water-use efficiency but a lower *A*.

The range and the response of *g*_m_ to temperature were reported to be markedly different among species (von Caemmerer and Evans, 2015). The *g*_m_ increased linearly in response to a temperature gradient ranging from 15 to 40°C for *Gossypium, Niocotiana*, and *Glycine*, culminating at 0.75 to 1 mol m^−2^ s^−1^ bar^−1^ (Bernacchi et al., 2002). However, for *Arabidopsis thaliana*, which is closely related to canola, the *g*_m_ remained unchanged over a temperature gradient, reaching a maximum of 0.22 mol m^−2^ s^−1^ bar^−1^ at 25°C and then decreasing. Similarly, reduced *g*_m_ was found under a high temperature, but there was no significant effect under drought. Previous studies have reported that *g*_m_ frequently, but not always, decreased in response to a water deficit (Flexas et al., 2008; Warren, 2008; Barbour and Kaiser, 2016). There is a strong relationship between *g*_s_ and *g*_m_ as the amount of CO_2_ in the sub-stomatal cavity should affect the fraction of CO_2_ reaching the chloroplast stroma through the *C*_i_, cell membranes, and cytoplasm (Olsovska et al., 2016). In general, drought triggers stomatal closure; consequently, mesophyll conductance would decrease. However, recent studies have shown that *g*_s_ and *g*_m_ conductance's can be uncoupled although environmental conditions might alter them in the same way (Barbour et al., 2016; Gago et al., 2016). Théroux-Rancourt et al. (2015) found that the *g*_m_ of hybrid poplar cuttings exposed to drought declined, but this response was delayed when compared to that for *g*_s_. In contrast, Barbour and Kaiser (2016) observed no effect on *g*_m_ under drought conditions when an adequate nitrogen supply was made available. The unchanged *g*_m_ under the moisture deficit in the current study might have been caused by a physiological acclimation in response to the stomatal closure and the decline of the *C*_i_, facilitating CO_2_ diffusion to the carboxylation site and preventing a shortage of substrate (Flexas et al., 2010). Such adjustment mechanisms are still unclear. They are more likely anatomical and morphological (i.e., the chloroplast position, leaf mass area; Milla-Moreno et al., 2016). Also, Momayyezi and Guy (2017, 2018) reported a substantial role for carbonic anhydrase in influencing *g*_m_.

Apart from the CO_2_ diffusion limitations, photosynthetic biochemical limitations have been reported to be remarkably heat-sensitive; however, drought has had a lesser effect (Flexas et al., 2006; Galmés et al., 2007). Indeed, previous studies have found that a severe water deficit has a limited effect or no effect on biochemical limitations rates (*V*_cmax_ and *J*) compared to its effect on stomatal limitations (Demirevska et al., 2009; Killi and Haworth, 2017). These findings are in agreement with the trends of non-significant effects of drought treatments on the *V*_cmax_ and the *J* but significant decreases to both under heat treatments. Under the heat + drought treatment, the *V*_*c*max_ and the *J* decreased further, showing the cumulative effects of the combined stressors; however, the *J*/*V*_cmax_ ratio did not change over the treatments (data not shown). Changes in the *J/V*_cmax_ ratio have been observed under adverse conditions. This could be the result of resource allocation, particularly nitrogen, to RuBisCO carboxylation or the electron transfer to optimize the photosynthetic assimilation rate (Hikosaka et al., 2006).

### Carbon and nitrogen-use efficiency

The results of this study showed that the Δ^13^C response to stress treatments paralleled the trends seen in the *g*_s_ response. The ratio of the *C*_i_ to the ambient CO_2_ fraction (*C*_i_/*C*_a_) is the main driver of the Δ^13^C variation in C_3_ terrestrial plants. This is influenced mainly by stomatal conductance (Farquhar et al., 1982). It is attributable to the shared path of the transpired H_2_O and the inbound CO_2_ fluxes, which stop in both directions when the stomata are closed in response to a water deficit. Several studies of plants in pots and in fields have demonstrated a strong relationship between water availability and the leaf carbon isotopic composition (Swap et al., 2004; Hartman and Danin, 2010; Cabrera-Bosquet et al., 2011). Given the environmental stability under which this experiment was conducted, the observed Δ^13^C variations were attributed solely to soil moisture availability. Under the optimal water supply, an increase in the air temperature did not affect the leaf Δ^13^C. However, Δ^13^C was lowest under heat + drought treatment compared to drought alone, suggesting a synergistic effect of the two stressors (“–* synergistic*” according to Piggott et al., 2015) which is supported by the significant H × D interaction effect on Δ^13^C (*P* < 0.001). An increased water use-efficiency (i.e., lower Δ^13^C) under heat + drought reflects a scenario that goes beyond the effects of single stresses, whereby a higher canopy temperature results from drought-induced stomatal closure in combination with heat treatment.

Several studies conducted in natural ecosystems along a rainfall gradient (in addition to a temperature gradient) showed higher δ^15^N values in C_3_ plants. In the current experiment, the δ^15^N values were influenced more by heat than drought. It is unclear, therefore, whether the variation in δ^15^N enrichment was the result of heat or drought or a combination of both. In contrast, in the natural stands, the δ^15^N values were higher in high nitrogen soils, and the nitrogen availability was higher in the warm and dry areas (Craine et al., 2009). Given the uniform soil characteristics and the short-term nature of this experiment, it is unlikely that heat and moisture deficits could influence the ^15^N vs. ^14^N fractions in the soil and, subsequently, the variations in δ^15^N observed in the leaves (Hartman and Danin, 2010). Therefore, it is proposed that the observed δ^15^N variation in the current experiment resulted from plant internal fractionation during the physiological processes that occurred during the nitrogen uptake, assimilation, allocation, and remobilization (Evans, 2001). In plant roots, the assimilation of nitrogen occurs for ${\text{NH}}_{4}^{+}$ through the glutamine synthetase–glutamate synthase (GS–GOGAT) pathway. However, the assimilation of ${\text{NO}}_{3}^{-}$ occurs in the roots and the leaves by nitrate reductase (NR) and the nitrite reductase (NiR) pathway, producing ${\text{NH}}_{4}^{+}$, which is subsequently assimilated through the GS-GOGAT reactions (Evans, 2001). Given that the δ^15^N of the leaves but not the roots was measured, the site of the nitrogen fractionation in *B*. *napus* remains inconclusive. Thus, a thorough understanding of *B. napus* metabolic pathways for the nitrogen uptake (${\text{NO}}_{3}^{-}$ and ${\text{NH}}_{4}^{+}$)-, assimilation-, allocation-, and remobilization-inducing δ^15^N variations would provide more information about plant nitrogen use, in turn a time-integrated measure of crop nitrogen-use efficiency.

### Seed yield

Heat stress during flowering was reported to reduce seed yield markedly by altering the gametogenesis (from meiosis to maturity), embryo sac differentiation, fertilization, and post-fertilization structures, such as the growth of the endosperm and the embryo (Wahid et al., 2007; Barnabás et al., 2008; Rieu et al., 2017), particularly in cool environment crops like *B. napus*. A higher sensitivity to heat for the female reproductive structures (ovary and embryo sac) than for the male structures was reported (Peet et al., 1998). In contrast, other studies have found pollen to be the most sensitive to heat (Saini and Aspinall, 1982). Drought also alters the reproduction and seed set in crops; however, the magnitude of this effect is generally less than that of heat. Drought stress lessens the available nutrients and photo-assimilation reserves that are essential for the development of reproductive structures (e.g., pollen tube elongation; see Barnabás et al., 2008).

As was hypothesized, the results showed a prevalent effect of heat, over that of drought, on yield as the seed weight of the plants exposed to heat reduced by 84% (vs. 31% for drought) and the silique number decreased by 43%. Angadi et al. (2000) observed that heat stress during the flowering stage, as opposed to the seed filling stage, had a pronounced effect on the *B. napus* yields. A threshold temperature close to 30°C during flowering has been reported as critical for yields for many herbaceous crops. The temperature threshold depends also on the plant species and the duration of exposure. For example, threshold temperatures range is 26°C for wheat (Stone and Nicolas, 1994) and 45°C for cotton (Ur Rahman et al., 2004), but for *Brassica* species, it is ~29.5°C (Morrison and Stewart, 2002). The results showed a considerable effect of heat at 29°C on seed yield, suggesting that the temperature threshold is much lower for canola (Gan et al., 2004; Aksouh-Harradj et al., 2006).

### Oil yield and composition

Oil is the most profitable product from canola seed processing, and its content and composition are affected by environmental factors (Jensen et al., 1996; Si et al., 2003). Seed oil stems mostly from photosynthetic carbon assimilation of leaves and green silique walls, later carbohydrates converted into triacylglycerol through a metabolic pathway occurring in the plastid, cytosol, and endoplasmic reticula (Baud and Lepiniec, 2010; and references therein). The effects of drought and heat on the oil content in oilseed crops have varied remarkably and have most likely been the result of a high G × E interaction (Pritchard et al., 2000; Sinaki, 2009; Zhang et al., 2014). Champolivier and Merrien (1996) reported a 6–12% decrease in oil content in the *B. napus* when the plants were subjected to a water deficit during flowering and silique development, but Aslam et al. (2009) reported a mere 3.2% reduction. In a different field study, Zarei et al. (2010) found no differences in canola oil content (an average of 37.27%) with or without irrigation. Similar to the observations made under drought conditions, Zhang et al. (2014) reported a significant effect of heat on oil content, which decreased by 52.5%. The results of this study were similar to those of Zhang et al. (2014). Heat, through its effect on the enzymatic panel involved in the lipid biosynthesis pathways, has been reported to decrease oil content (Iyer et al., 2008; Baud and Lepiniec, 2010).

In addition, the silique walls, along with the leaves during the post-flowering stages, are significant sources of photosynthates (Aschan and Pfanz, 2003; Bennett et al., 2011; Hua et al., 2012). Thus, abiotic stressors during flowering would affect silique development and subsequently reduce the available photo-assimilates for triacylglycerol biosynthesis and oil accumulation in the seeds. In addition, oxygen availability in silique was also cited as a limiting factor in seed development (Porterfield et al., 2000). Vigeolas et al. (2003) reported that a low oxygen content in *B*. *napus* seeds resulted in reductions in the adenosine triphosphate (ATP) level and the triacylglycerol content. Similarly, Rolletschek et al. (2007) observed a negative correlation between the ambient temperatures and the oxygen levels in sunflower seeds, a relationship that affects oil composition.

As was hypothesized, heat noticeably altered the oil profile, but drought had a marginal effect. Previous studies on heat have reported changes in the oil composition, particularly the fatty acids, and protein content in oil seed crops (Dornbos and Mullen, 1992; Flagella et al., 2002; Wang and Frei, 2011). This effect has been attributed to the enzymes involved in biosynthesis and the conversion of fatty acids in various cellular compartments (Flagella et al., 2002; Di Caterina et al., 2007; Hernández et al., 2009). For example, Martínez-Rivas et al. (2003) found that in sunflowers, the activity of oleate desaturase, an enzyme involved in the desaturation of fatty acids in oilseeds, was altered by heat. Moreover, oil composition has been shown to be influenced by the action of abiotic stressors on the transport of fatty acids through various organelles, particularly from plastids to cytosol, where oleic acid (18:1) is converted into linoleic acids (18:2) and linolenic acids (18:3) (Browse and Somerville, 1991). In general, the fraction of polyunsaturated fatty acids decreased; however, the fraction of saturated fatty acids and, concurrently, oleic acid (mono-unsaturated) increased in response to stressors (Pritchard et al., 2000; Wang and Frei, 2011). The results of the current study were similar to those previously seen for heat conditions, which increased the proportion of saturated fatty acids, but not for drought, which increased the relative content of unsaturated fatty acids (Aslam et al., 2009).

Along with triacylglycerol, seed proteins represent a major form of energy reserves in the *Brassica* species, and their respective contents are negatively correlated (Grami et al., 1977; Jensen et al., 1996). Thus, stressors decreasing the oil content in seeds would concurrently increase the protein fraction (Henry and MacDonald, 1978; Rossato et al., 2001; Rathke et al., 2006). Overall, the results of this study are in agreement with these previous findings although the drought treatment increased the seed proteins without affecting the oil content. For the most part, heat exceeded the effect of drought in augmenting the seed protein content (e.g., heat shocks proteins as chaperones; Kotak et al., 2007) but not the osmoprotectants (polyamine, glycine betaine, and proline; Singh et al., 2015). Under the heat treatment, the well-watered plants might maintain the optimal nitrogen uptake and accumulation in the vegetative parts, subsequently nitrogen remobilized from the senescent tissue to the seeds. Given that the seed number was considerably reduced by heat, the nitrogen supply could have been superior to the demand, thus boosting the seed protein content. Lohaus and Moellers (2000) demonstrated that the external nitrate supply is determinant of the total amino acid content of the phloem sap of leaves and is positively correlated with the seed protein content in two *B*. *napus* cultivars. The partitioning of the oil and protein content was under G × E control, and the molecular basis of this trade-off is still unclear (Si et al., 2003; Chao et al., 2017).

## Conclusions

Overall, the results of this study showed a divergence between the effects of drought, heat, and heat + drought on canola seed yield and oil quality. Drought affected the carbon assimilation rate mainly through the limitation of CO_2_ diffusion through the stomata and the seed yield components. The effects of the heat conditions were clearly manifested in the alteration of the reproductive organs and process, leading to a substantial reduction in the seed yield and the number of siliques. To a lesser extent, heat impaired the internal CO_2_ diffusion and the RuBisCO carboxylation and regeneration. This was most likely the result of thermal damage to the enzymes involved in photosynthetic assimilation. Similarly, heat had a prevailing effect over drought on seed composition, which is greatly influenced by the conversion and transport of photo-assimilates to the seeds, in turn higher levels of saturated fatty oils. Such higher levels of saturated fat under warmer climates could affect industrially relevant traits: the taste, freshness, and shelf life of canola oil. The adverse effects of moderate to severe drought can be mitigated by irrigation and/or using genotypes with greater water-use efficiency. However, heat requires the breeding of heat tolerant canola as a major tool to manage the harmful effects of such environments. Such breeding efforts could target the carboxylation capacity and diffusion of CO_2_ along the mesophyll pathway as well as the tolerance of the reproductive organs for elevated temperatures.

## Author contributions

RS and RE conceived and designed the experiment. RE conducted the experiment, analyzed data, interpreted results, and drafted the manuscript.

### Conflict of interest statement

The authors declare that the research was conducted in the absence of any commercial or financial relationships that could be construed as a potential conflict of interest.
