# Supplementary material for: Canola Responses to Drought, Heat, and Combined Stress: Shared and Specific Effects on Carbon Assimilation, Seed Yield, and Oil Composition
Source: Front Plant Sci. 2018 Aug 30;9:1224. doi: 10.3389/fpls.2018.01224 (PMC6125602; doi:10.3389/fpls.2018.01224)
Supplement: Supplementary file 1 [file Table_1.docx]

**Table S1.** Summary of the topsoil characteristics. OM refers to the organic matter content and EC to the electrical conductivity of the soil water extract.

|  | **pH** | **OM**  (%) | **EC**  (mmhos.cm^-1^) | **Nitrate-N** | **NH4** | **P (Olsen)** | **K**  (ppm) | **Ca** | **Mg** | **Na** | **Cl** | **CEC**  (meq/100g) |
| --- | --- | --- | --- | --- | --- | --- | --- | --- | --- | --- | --- | --- |
| **Mean** | 7.9 | 1.9 | 0.5 | 5.2 | 3.8 | 23.2 | 295.4 | 3846.2 | 433.4 | 32.8 | 31.4 | 23.8 |
| **± SD** | ± 0.07 | ± 0.16 | ± 0.08 | ± 1.72 | ± 0.61 | ± 1.91 | ± 20.3 | ± 156.2 | ± 19.4 | ± 3.7 | ± 2.8 | ± 0.95 |
