# Supplementary material for: Canola Responses to Drought, Heat, and Combined Stress: Shared and Specific Effects on Carbon Assimilation, Seed Yield, and Oil Composition
Source: Front Plant Sci. 2018 Aug 30;9:1224. doi: 10.3389/fpls.2018.01224 (PMC6125602; doi:10.3389/fpls.2018.01224)
Supplement: Supplementary file 2 [file Image_1.pdf]

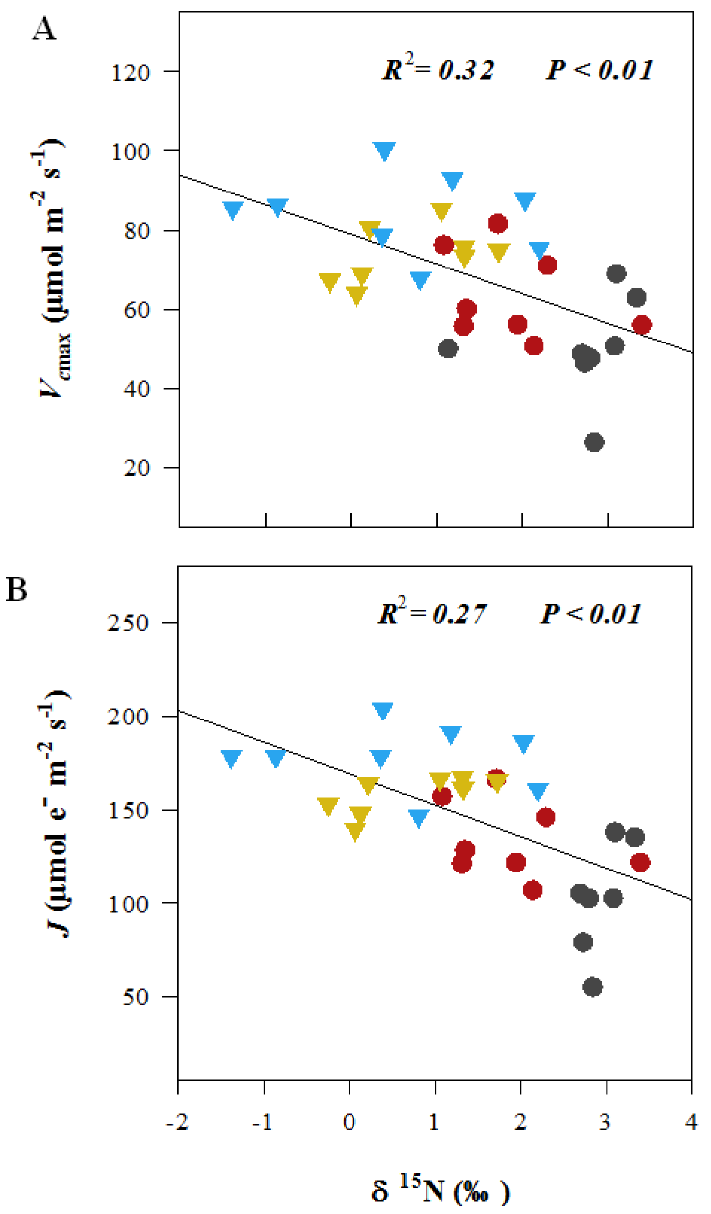

FIGURE S1. The relationships between the leaf nitrogen isotopic composition ($\delta^{15}N$) and the maximum carboxylation rate of ribulose-1,5-bisphosphate carboxylase/oxygenase (V_{cmax}, **A**) and between $\delta^{15}N$ and the photosynthetic rate of electron transport (*J*, **B**). ▼: well-watered plants (WW, 23 °C); ▼: drought (23 °C); ●: heat (29 °C); ●: heat+drought (29 °C). The lines were fitted by regression using all the points in the plot.
