# Supplementary material for: Canola Responses to Drought, Heat, and Combined Stress: Shared and Specific Effects on Carbon Assimilation, Seed Yield, and Oil Composition
Source: Front Plant Sci. 2018 Aug 30;9:1224. doi: 10.3389/fpls.2018.01224 (PMC6125602; doi:10.3389/fpls.2018.01224)
Supplement: Supplementary file 3 [file Image_2.pdf]

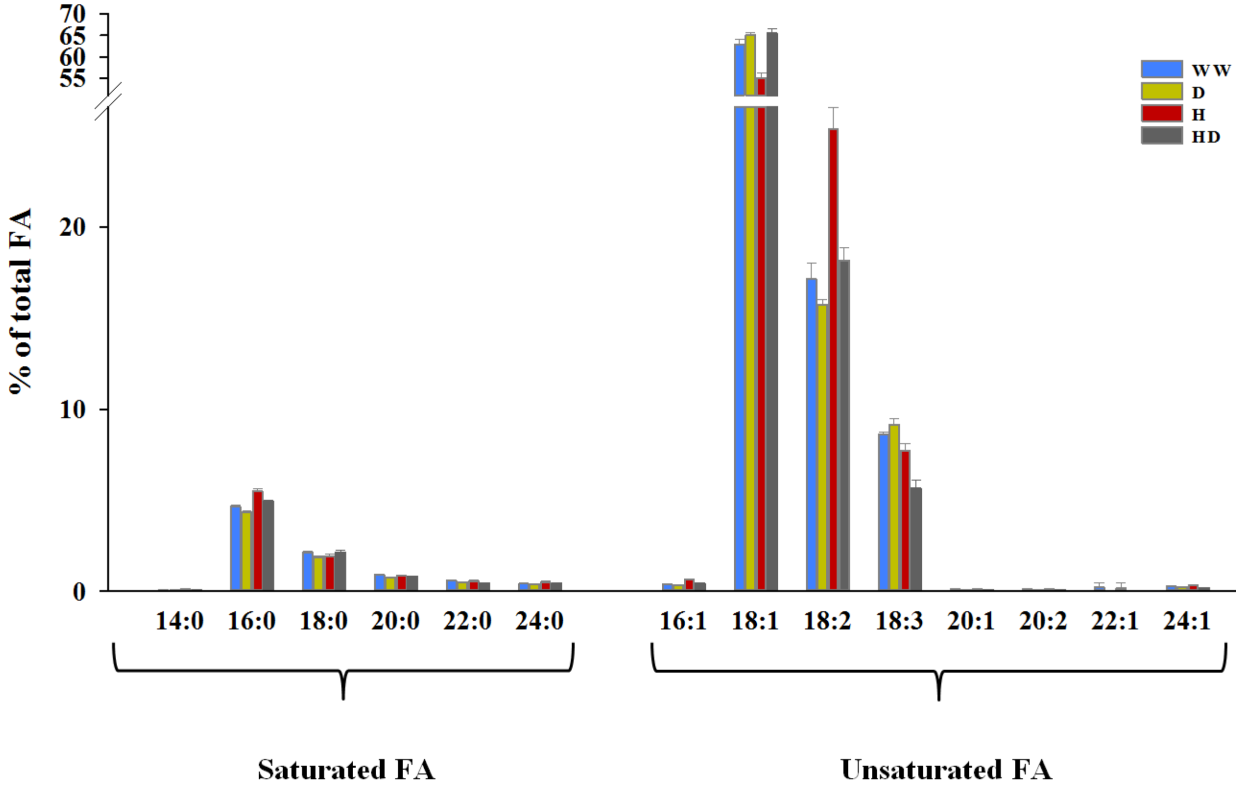

FIGURE S2. The total percentage of seed fatty acids (FA) under the different treatments.
14:0: myristic acid; ***16:0***: palmitic acid; ***16:1***: palmitoleic acid; ***18:0***: stearic acid; ***18:1***: oleic acid; ***18:2***: linoleic acid; ***18:3***: α-linolenic acid; ***20:0***: arachidic acid; ***20:1***: gondoic acid; ***20:2***: eicosadienoic acid; ***22:0***: behenic acid; ***22:1***: erucic acid; ***24:0***: lignoceric acid; ***24:1***: Nervonic acid.
